# Recent Development in Metasurfaces: A Focus on Sensing Applications

**DOI:** 10.3390/nano13010118

**Published:** 2022-12-26

**Authors:** Nikolay L. Kazanskiy, Svetlana N. Khonina, Muhammad A. Butt

**Affiliations:** 1 IPSI RAS-Branch of the FSRC “Crystallography and Photonics” RAS, 443001 Samara, Russia; 2 Samara National Research University, 443086 Samara, Russia

**Keywords:** metasurface, metamaterial, hybrid metasurface, all-dielectric metasurface, all-metallic metasurface, photonic sensor, perfect absorber

## Abstract

One of the fastest-expanding study areas in optics over the past decade has been metasurfaces (MSs). These subwavelength meta-atom-based ultrathin arrays have been developed for a broad range of functions, including lenses, polarization control, holography, coloring, spectroscopy, sensors, and many more. They allow exact control of the many properties of electromagnetic waves. The performance of MSs has dramatically improved because of recent developments in nanofabrication methods, and this concept has developed to the point that it may be used in commercial applications. In this review, a vital topic of sensing has been considered and an up-to-date study has been carried out. Three different kinds of MS absorber sensor formations, all-dielectric, all-metallic, and hybrid configurations, are presented for biochemical sensing applications. We believe that this review paper will provide current knowledge on state-of-the-art sensing devices based on MSs.

## 1. Introduction

Metamaterials (MMs) are manmade electromagnetic (EM) materials with periodic arrangements of metallic structures that are smaller than the wavelength of the incident EM wave [1]. Additionally, the materials have exotic electrical and magnetic properties that are not commonly found in nature, for instance, backward propagation [2], the reverse Doppler effect [3], Vavilov-Cerenkov effect [4], negative refraction [5], diffraction-limit breaking imaging [6], cloaking [7], etc. They can also exploit EM-wave beams in unexpected ways [8].

Furthermore, MMs can strongly localize and intensify fields, making them effective for detecting exceptionally small concentrations of analytes as well as improving sensor selectivity for detecting nonlinear chemicals [9]. Numerous new or enhanced applications of MMs have lately been formulated based on this feature. For instance, it has been suggested to enhance the sensing performance of surface plasmon resonance (SPR) sensors by replacing the metal components with MMs [10]. The use of MMs as high-frequency sensors has also been considered [11]. The performance of MM sensors is discussed in [12], and the findings indicated that MMs can significantly improve the sensitivity and resolution of the sensors. These provide for additional levels of freedom in the sensor model, which promises to increase sensitivity and simplify readout [13,14,15].

Mechanical or electronic devices have fundamentally altered the way we work, communicate, amuse ourselves, and travel in the modern world. Human–machine interface (HMI) technologies are widely used in our daily lives, whether or not they are regarded as such. Only when the machines can react to human directions for completing desired activities can they detect the environment through a variety of signals. MMs have provided an excellent foundation for the development of highly accurate sensing materials and devices from several disciplines, opening up a wide range of HMI application possibilities. Significant advancements have been made in this area during the past ten years [16]. An in situ trainable real-time digital metasurface (MS) imager that can provide the radiation patterns needed for machine learning optimized measurement modes has been proposed. This imager is electrically reprogrammed in real-time to obtain the best possible outcome for all the data, enabling the storage and transmission of full-resolution raw data in constantly changing settings [17]. With the use of a single physical hardware imager that was real-time reprogrammed, high-accuracy image coding and identification were shown for a variety of picture sets, including through-wall body movements and handwritten digits. The electronically controlled MS imager creates new opportunities for effective surveillance, quick data collection and processing, imaging at varied frequencies, and more.

The effectiveness of functional optical devices like lenses, holograms, wave plates, spectrometers, etc. is constrained by the intrinsic optical losses that plasmonic MSs experience due to significant electron-electron and electron-phonon scattering in metals [18,19]. All-dielectric MSs have been created in the search for extremely effective planar optical manipulators. Dielectric MSs, in contrast to plasmonic (or all-metallic) MSs, are based on the collective light scattering (sometimes referred to as Mie scattering) of the fundamental high-index dielectric nanoparticles with sizes similar to the wavelength of light within the particles [20,21,22,23,24].

All-dielectric MSs can reduce optical loss because the large bandgap energy of dielectric materials restricts optically generated interband transitions [25]. Because there are no free charge carriers present when light with sub-bandgap energy interacts with dielectric nanoparticles, displacement currents rather than conduction currents are created. In contrast to metallic nanoparticles, where a substantial percentage of the incident optical energy reverts to heat and strong electric field concentration occurs adjacent to the surface outside the nanoparticles, this leads to insignificant optical losses and extraordinary E-field concentration inside the dielectric nanoparticles [26]. All-dielectric MSs greatly outperform plasmonic MSs in efficacy and resonance quality factor due to the low-loss characteristic [27].

**Figure 1:**
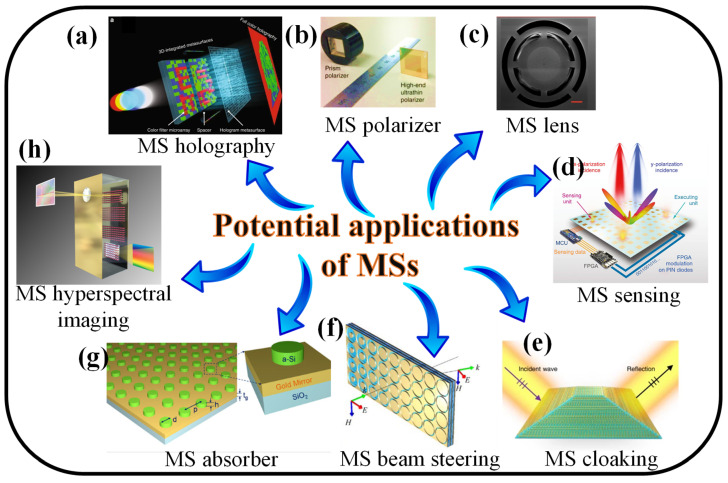
Some of the potential applications of MSs, (**a**) holography [31], (**b**) polarization control [32], (**c**) Lenses [33], (**d**) Sensors [34], (**e**) cloaking [35], (**f**)beam steering [36], (**g**)absorbers [37], and (**h**) hyperspectral imaging [38].

Due to their capacity to generate superior quality-factor (Q-factor) resonances, MS analogs of electromagnetically induced transparency (EIT) have come into the spotlight in recent years in the nanophotonics community. EIT is a phenomenon wherein a material’s optical response is governed by an EM-field. As predicted, the absorption of three-level atomic ensembles is nullified in the presence of an auxiliary EM-field, and this is first demonstrated in the gas-phase atomic medium [28]. The phenomenon is caused by the quantum interference effect, in which the transition amplitudes of several paths destructively interact with one another. Numerous optical and quantum information processing applications have been made possible by the EIT effect. For example, applications for low-loss slow-light systems and extremely sensitive optical sensors are anticipated to benefit from these resonances. However, the performance of devices is severely hampered by the achievable Q-factors in typical plasmonic EIT MSs, which are only 10 or lower due to ohmic losses. An EIT-equivalent utilizing silicon-based all-dielectric MSs is presented in [27]. A Q-factor of 483, along with coherent interaction between nearby meta-atoms, results in an RI sensor with a figure-of-merit of 103 and extraordinarily low absorption loss. The potential applications of MSs, such as holography, polarization control, lenses, sensing, cloaking, beam steering, perfect absorbers [29], and hyperspectral imaging, are presented in Figure 1. However, in this review, we aim to discuss the sensing applications of MSs in detail. Optical sensors can identify, analyze, and quantify molecules for a variety of applications by using different types of light-matter interactions. An optical sensor is made up of a light source that produces EM waves, a sensing platform where light interacts with matter, and a detector that recognizes and measures EM-wave spectrum alterations in response to targeted analyte contact. An optical sensor’s sensing concept is based on changes in an optical platform’s signature optical signal brought on by interactions with analyte molecules, which are subsequently converted into quantitative and/or qualitative measurements. The engineering and design of the sensor platform are critical because they define the interplay of light and matter [30].

There are many different types of EM sensors described in the literature, including interferometers [39], waveguides [40,41], gratings [42,43,44], cavities/resonators [45,46,47], SPR-based structures [48], and, most recently, all-dielectric MMs [49]. They exhibit some benefits, but they also have several downsides and restrictions, including electrically massive structures, high losses, dispersive behavior, polarization dependency, and difficulty in manufacturing [50]. The 2D variants of MMs, known as MSs, play an important role. In the literature, several interesting reviews on MSs can be found which are highly recommended to readers. For instance, in [51], the most recent developments in material-specific surface functionalization and texturing are discussed as they are applied to sample optical MSs. Detailed instructions for the widespread implementation of this approach are also provided, along with an understanding of the underlying chemistry that powers functionalization and texturing operations. Overall, a clear and comprehensive manual for altering MSs with a focus on sensing applications is provided. In [52], the most recent research on EM-MSs and their emerging uses in sophisticated integrated instruments and devices, from design to physical implementation, is presented. The analytical coupled-mode theory model and widely used building blocks for creating functional MSs are included in the design methodology. The current advancements in different sensors based on MMs and MSs are presented in [53]. Last but not least, in [54], the most recent developments in MSs for different bioapplications are discussed. These comprise magnetic resonance imaging (MRI), quantitative phase imaging, optical chiral imaging, endoscopic optical coherence tomography (OCT), fluorescence imaging, and super-resolution imaging (QPI). Applications of MS in biosensing, such as the detection of DNAs, cells, and cancer biomarkers, are described. In order to serve as a reliable reference for future efforts in this fascinating and quickly developing field, the study presented in [55] addresses MMs and MSs in great detail from the viewpoints of materials, mechanisms, and advanced metadevices.

MS-based devices have been categorized as narrowband [56,57], broadband [58], and wideband [59], depending on the materials used, their morphology, and their frequency responses. The classic example of a narrowband operation is Fano resonance [60]. One of the most crucial features of Fano resonance is the acute resonance peak coupled with high local field augmentation. It serves as the basis for several real-world applications, including nonlinear photonics and biological sensing [61]. High-performance color filters [62], broadband absorbers [58], and polarizers [32,63], as well as super-resolution imaging beyond the diffraction limit, may all be supported by MS dispersion for multiband and broadband applications [64].

The use of EM fields and waves to create sophisticated sensing and medical diagnostic devices has lately gained popularity [65]. Affected tissues frequently undergo structural, metabolic, and mechanical alterations, which strongly suggest that their EM characteristics have changed [66]. An optical sensor’s primary objective is to detect these variations: the disease causes changes in the sample’s EM properties (specifically, refractive index or absorption), which the sensor recognizes in terms of frequency position, and the magnitude and bandwidth on its output signal. In this sense, micro- and/or nano-structures have been applied as sensing platforms in the fields of biology, environment, safety, and medicine [67]. EM sensors provide benefits over conventional methods when it comes to the variety of materials and samples that may be studied, including speed, affordable, high sensitivity, selectivity, and label-free analysis [68,69].

**Figure 2:**
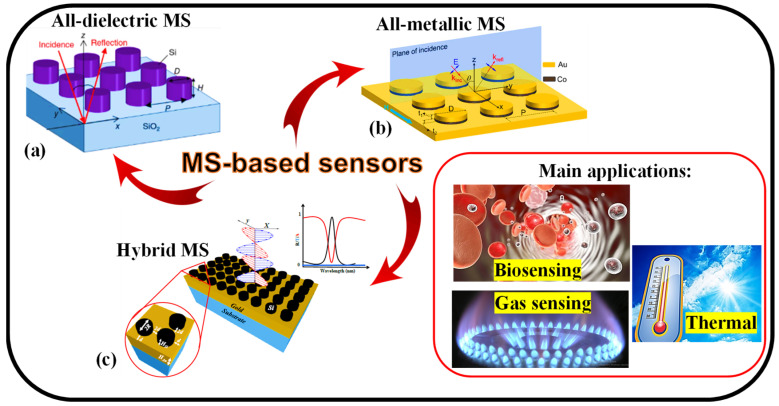
Types of MS such as (**a**) All-dielectric MS [81], (**b**) All-metallic MS [82], and (**c**) Hybrid MS [83], for biosensing [84], gas sensing [85], and temperature sensing applications [86].

Refractive index (RI) biosensing is extremely practical and a typical application of MMs and MSs [70]. As a result of biomolecular interactions in analyte layers, the RI changes. Because of their special ability to perform sensitive and label-free biochemical tests, EM RI sensors can be employed in several chemical and biological sensing applications [71]. By manipulating specific meta-atoms and their configurations, it is likely to significantly alter the resonant EM spectrum that is ruled by the ambient medium. The output spectrum, which is utilized to calculate the RI of the nearby biomolecular analytes, can vary due to this resonant feature. As a result, certain wavelengths and sensitivity levels must be built into mass configurations. Furthermore, there are several benefits that RI sensors implemented on MM- and MS-based sensing platforms [72] have over traditional surface plasmon polariton (SPP)-based biosensors [73,74,75]. As RI change is sensed through macroscopic optical responses, primarily reflection or transmission of focused input beams, MM- and MS-based RI sensing devices work better than SPP-based sensors, largely due to manufacturing tolerance and signal stability [57,76]. In this paper, three different kinds of structures known as all-metallic, all-dielectric, and hybrid MSs are discussed for sensing applications as illustrated in Figure 2. 

Plasmonic (all-metallic and hybrid) MSs have developed steadily and have found use in several optical components. Silver and gold are the two substances that are most frequently utilized for plasmonic MSs [77,78]. When viewed as potential components for useful technologies, these materials have several drawbacks. They cannot be used for activities that need CMOS compatibility, ultrathin films, chemical inertness, high-intensity laser resistance, or temperature stability. Emerging transition metal nitrides are crucial in this area of plasmonics application. Because of their great biocompatibility, electrical conductivity, chemical and thermal stability, and similarity to valuable metals in terms of their electronic structure, transition metal nitride compounds are of particular interest.

An ideal substitute plasmonic material would be less expensive to produce, have a higher melting point, be more chemically stable, and be capable of being chemically synthesized using current nanofabrication techniques. This is the case with group IV transition metal nitrides (TMNs), which are refractory materials, with exceptionally high melting temperatures and good chemical stability. In the visible-near-IR range, TMNs also exhibit a comparable plasmonic response to silver and gold [79]. Other group IV TMNs, such as zirconium nitride and hafnium nitride, remain undeveloped despite titanium nitride’s reputation as a potential tool for plasmonic applications in the visible and NIR. Nevertheless, the few linked earlier investigations have shown that hafnium nitride has better plasmonic efficiency for photothermal applications relative to gold and even titanium nitride [80].

## 2. Requirements for Sensor Designing

In a variety of sectors, together with biochemical recognition, environmental toxic examining, and security assessment, optical sensing is a potent approach for identifying analytes [87]. Target analytes like proteins, lipids, and DNA have enticed increasing interest in the detection and differentiation of target analytes due to nondestructive and label-free optical sensing’s capacity to detect molecular concentrations and interaction kinetics without interference from fluorescent labels or other external tags [88]. Nevertheless, due to the mismatch between wavelengths and molecule sizes, the sensitivity for nanometer-scale and trace-amount analytes is relatively low. To control EM waves in the microwave, terahertz, and optical spectrums, MS—a device made up of several subwavelength nanoresonators—can be utilized [89].

The four factors listed below can determine whether a sensing device can detect a slight change in the ambient medium: To prevent background and substrate absorption, the sensors’ operating frequency must be as low as possible. As a result of their small surface area and tendency to operate more frequently, conventional sensing devices provide a substantial problem in this regard. Because of this, it is necessary to keep the sensor’s layout compact while also minimizing its operating frequency. Secondly, to precisely track the shift in transmission spectra, the sensors must generate a robust and detectable readout signal with a sharp resonant characteristic. The linearity of sensing, which is correlated with the Q-factor of sensors, is the subject of the third criterion; and sensor sensitivity is the fourth requirement. When the sensitivity is significantly high, it is simpler to distinguish tiny shifts in the spectra following the externally applied load when there are fewer data points in one frequency scan of the network analyzer.

Resonant micro- and nano-photonic RI sensors have been used for a long time to analyze chemical and biological samples in real-time without the use of labels, such as to identify target biomolecules in a biological fluid or find organic liquid components [90,91]. The light-matter interaction causes the sensor resonance frequency to change when target molecules encounter light [92]. The frequency shift is then determined and used to identify target molecules. The sensitivity (S) and figure of merit (FOM) are depicted as follows to assess the sensing capacities of the device as shown in Equation (1) and Equation (2), respectively:

(1) $$S=\frac{∆\lambda }{∆n};$$

(2) $$\mathrm{FOM}=\frac{S}{\mathrm{FWHM}};$$

where ∆λ is the change in resonance wavelength concerning the change in ambient refractive index (∆n). FWHM is the full width at half maximum of the resonance wavelength. 

## 3. Working Mechanism of the MS-Based Sensors

**Figure 3:**
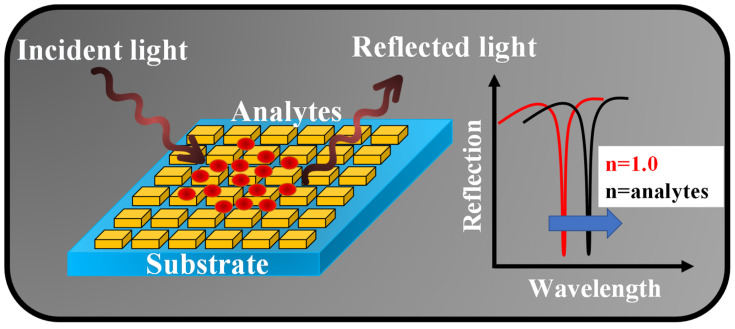
Sensing mechanism of an MS device based on the wavelength interrogation method.

Utilizing three various aspects of light, such as intensity, phase, and wavelength approaches, one may see the transmission or reflection spectra of sensor devices. Using the wavelength interrogation method, the suggested sensor device may be described. The most typical inspection technique, which is frequently employed in resonant structures, involves measuring fluctuations in the resonant wavelength [75]. When the refraction index of the ambient medium changes, it results in the transformation in the effective refractive index of the resonant mode, as a result, a shift in the resonant wavelength is observed. Optical systems that rely on wavelength interrogation frequently use a polychromatic light source, such as a halogen lamp or a superluminescent diode, that covers the whole spectrum where the resonance wavelength is predicted to appear [93]. When the fixed incidence angle setup and wavelength interrogation technique are used, halogen lamp technology is preferred because it is significantly good concerning the broad spectrum of light. Figure 3 (left) shows the schematic of an MS-based sensor where broadband light is incident on it at a certain angle and the reflected light is collected via a spectrum analyzer. The reflection spectrum (right) shows a resonance dip at a certain wavelength that performs a redshift when the analytes under observation are placed on the MS. 

## 4. MS-Based Perfect Absorbers for Sensing Applications

For many years, perfect absorbers have been a hot topic within the field of MMs. Although the first realization of a perfect absorber was reported more than 30 years ago, research is still being conducted to show and create perfect absorbers with new and creative methods [94]. Broadband and narrowband perfect absorbers offer a wide range of practical uses. A polarization-independent MM-based broadband absorber with a maximum absorbance of 95% was recently reported [95]. Additionally, four distinct multiband MS absorbers with split-ring resonators have been described, with a maximum absorbance of 99.9% in the THz range [96]. A metal-dielectric (Cr–Si) MS absorber with a wide operating bandwidth and a large incidence angle (70°) has been described [97]. With a maximum absorbance of 90%, another broadband wide-angle polarization-insensitive plasmonic MS absorber has been found [98]. The absorbing operation for both directions of the incident light has been reported to be increased by a bi-directional plasmonic MS absorber with a free substrate [99]. Along with the wide-angle, broadband, and polarization-independent performance, the adjustable absorbance feature is another intriguing phenomenon because of the variety of uses it has. MS absorbers that can be adjusted fluidically [100] and electrically [101] have recently been described.

By adding dielectric material into the designs, one of the key goals is to solve the drawbacks of metallic absorbers. This will reduce losses from ohmic losses, heat production in the metal, and the activation of surface plasmons at higher frequencies. Ultrabroadband MSs may make it possible to develop optically thin coatings with the desired spectral characteristics over wide spectrum ranges, opening the door to applications including structural coloration, radiative heat management, and camouflage. Conversely, very narrowband absorbers can be used to create extremely sensitive temperature sensors or emitters [102].

**Figure 4:**
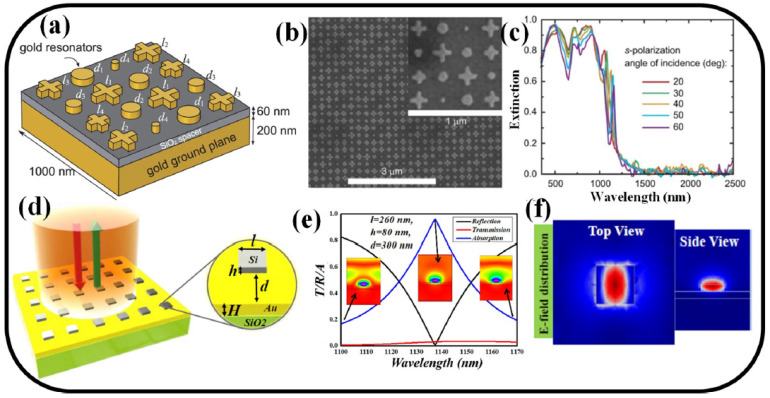
(**a**) Schematic representation of a broadband MS solar absorber [58], (**b**) SEM image of the fabricated device [58], (**c**) Experimentally measured extinction graph versus wavelength [58], (**d**) schematic of a hybrid narrowband perfect absorber [57], (**e**) Transmission/reflection/Absorption spectrum [57], and (**f**) E-field distribution at the resonance wavelength [57].

Apart from transmission- and reflection-based sensors [60,103,104,105], recent years have seen a rise in interest in the investigation of MS-based perfect EM-wave absorption for RI sensing applications [106,107]. Magnetic resonance can cause a sizable magnetic dipole to intermingle with the magnetic field of incident EM waves in artificial MSs, leading to the creation of an effective permeability [57]. The incident EM waves will be almost entirely absorbed at a specific frequency range when the impedance of artificial MSs is matched with that of a vacuum. Considering their absorption bandwidths, ideal absorbers may be broadly divided into broadband absorbers [58] and narrowband absorbers [72] in practical applications. It is shown that a metallic MS architecture can support a wide-angle, polarization-independent, broadband absorber that achieves greater than 90% absorptance in the visible and NIR range of the solar spectrum while having low absorptivity (emissivity) at the mid-and far-infrared wavelengths. Eight pairs of gold nanoresonators make up the complicated unit cell of the MS solar absorber, which is set apart from a gold ground plane by a thin SiO_2_ spacer as illustrated in Figure 4a. The SEM image of the fabricated sample is illustrated in Figure 4b. The experimental studies showed high-performance absorption for both s- and p-polarizations over a broad range of incidence angles [58]. Figure 4c depicts the observed absorptance for both s- and p-polarizations at a 20° angle of incidence, showing the strong absorption that is polarization-independent and covers practically the full solar spectrum [58].

The well-known SPRs or other resonance modes for metal nanostructures can provide a significant EM field augmentation that can be used for accurate absorption and RI monitoring. Shi et al., for instance, theoretically investigated a multi-band perfect absorber for visible and NIR sensing applications that are established on the excitations of localized surface plasmons, delocalized SPPs, and lattice plasmon resonances in the periodic arrays of gold nanodisks with prismatic holes standing on silica/gold bilayer films [108]. Nanostructured graphene has an effective permittivity of the typical Drude model, it can sustain SPRs from MIR to THz frequencies [109]. In comparison to those in metal nanostructures, the plasmon resonances of graphene nanostructures have smaller bandwidths because of their low damping rates, which leads to higher EM field confinement [110]. More crucially, a bias voltage may be used to control the locations of the plasmon resonances in graphene [111]. These characteristics make nanostructured graphene a strong contender for applications in perfect absorption and sensing.

A numerical analysis of a narrowband perfect hybrid MS absorber is provided in [57]. On top of an 80 nm thick gold layer is the periodic array of silicon meta-atoms as illustrated in Figure 4d. The silicon meta-atoms are employed to activate the surface plasmon by scattering light through the gold layer, which blocks the broadband light at normal incidence. The transmission/reflection/absorption spectra and the E-field distribution at resonance wavelength are illustrated in Figure 4e and Figure 4f, respectively [57]. Because the impedances of the electric and magnetic dipoles are perfectly matched, a maximum absorption of 95.7% is attained at a resonance wavelength of 1137.5 nm. Wide-angle incidence spanning from 0 to 80° has little effect on absorption. The suggested MS device is thought to have applications in solar photovoltaic and biochemical sensing [57].

## 5. Types of MS-Based Sensors

MSs have become prominent as potential solutions to the problem of heavy optical components. To effectively regulate phase, polarization, and emission, MSs provide a fundamentally novel approach to light control based on scattering from resonant nanostructures rather than traditional refraction and propagation [112]. Regarding active creation, manipulation, and recognition of light for quantum technologies, holography, and sensing, this viewpoint highlights state-of-the-art MSs and offers a road map for potential developments.

Perfect absorbers have long been a popular topic in the field of MMs. It has been 45 years since the first demonstration of a perfect absorber was published [94]. Since then, MS-related research has grown significantly, and fresh methods for creating ideal absorbers with top-notch specifications are continually being created and tested. The most basic representation of a perfect absorber is a uniform absorbing substance near air. The absorbing medium must have absorption, the imaginary portion of the RI, and a wave impedance of unity to match the impedance of air [113]. Reflection will always happen if there is an impedance mismatch, which is problematic for a perfect absorber. To obtain a perfect absorber with the absorption of unity, transmission and reflectivity must be concurrently minimized. Simply decreasing transmission may be accomplished by using a metallic backplate [57].

In recent years, it has been shown that EM resonance can also be controlled using dielectric MMs, which are made of dielectric microstructures. Dielectric MMs offer an optimal resolution for reducing the absorption bandwidth due to their special advantage of minimal loss [114]. Additionally, recent reports utilize dielectric microstructures for ultra-narrowband absorbers [115,116]. Nevertheless, there is still room for improvement in the sensing capabilities of a dielectric MM absorber. 

An investigation of a NIR ultra-narrowband absorber uses metal substrates covered in ultra-sparse dielectric nanowire grids [107]. The modeling findings demonstrate that the absorber has an FWHM of 0.38 nm and an absorption rate of more than 0.99. The field distribution also shows that the low loss in the guided-mode resonance is the source of the ultra-narrowband absorption. The absorber has a high S of 1052 nm/R.I.U and a large FOM of 2768, which means that this ultra-narrowband absorber can be utilized as a high-performance RI sensor. These characteristics are made possible by the ultra-narrow absorption bandwidths and the E-field that is primarily distributed out of the ultra-sparse dielectric nanowire grids [107].

A thorough investigation of the hybrid MS perfect absorber (HMSPA) established on square meta-atoms (S-MAs) and hollow square meta-atoms (HS-MAs) sensing properties is described in [76]. The fact that both models may offer >90% absorption in the narrowband region makes them suitable for filtering purposes. For biosensing applications, the HMSPA with HS-MAs is superior to S-MAs because it is more sensitive to minute changes in the RI of the surrounding medium. The S-MA-based HMSPA has a sensitivity of about 135 nm/R.I.U, which can be additionally improved to 355 nm/R.I.U by employing HS-MAs. Additionally, using the suggested device for temperature sensing is made possible by depositing a temperature-sensitive substance on the MS. The HS-MA-based HMSPA has a temperature sensitivity of −0.18 nm/C for the temperature range of 20 °C to 60 °C thanks to the exceptional thermo-optic coefficient of polydimethylsiloxane (PDMS). With their simplicity in device fabrication and strengths in light coupling, the suggested HMSPA structures have the potential to be beneficial in filtering, biochemical sensing, and thermal-sensing uses [76].

### 5.1. All-Metallic MS-Based Sensors

Traditional MM systems often have a poor Q-factor because of the significant radiation loss and absorption. An effective solution to the problem is provided by Fano resonances in ultrathin MSs. In rectangular-hole dimers with broken symmetry, the trapped-mode resonance is investigated [117]. The asymmetric hole dimers on freestanding metal screens offer an all-metallic platform for modeling the high-Q resonances in contrast to the well-studied asymmetric particle dimers. A Q-factor of 200 has been greatly raised by the tests in the microwave region. According to numerical models, the hole E-field (and intensity) may be enhanced by 127 times (and 16,000 times) in contrast to the incident field. Additionally, a thorough investigation of the trapped-mode resonance’s evolution characteristics concerning structural factors has been conducted [117].

For most early MS applications, traditional plasmonic materials like gold and silver have traditionally been employed. Since it can be made relatively easily, evaporated silver is frequently employed in MSs, although it tends to be lossier because of electron scattering at grain boundaries. Bulk silver has strong plasmonic characteristics in the visible frequencies. Additionally, because gold and silver tend to form nanoislands rather than homogeneous films at thicknesses below 10 nm, growing ultrathin (on the scale of a few nanometers) gold and silver films is inherently difficult. As far as fabrication goes, gold and silver are incompatible with complementary metal-oxide semiconductor (CMOS) technology because gold is easily diffused into the substrate and silver has poor chemical stability. Furthermore, the melting temperatures of these noble metals are modest. As a result, at high temperatures, noble metal nanostructures are easily deformed. Noble metals cannot be used in high-temperature applications as a result, which presents a problem for plasmonics because plasmon oscillations always lead the metals to get highly heated, at least within the confines of the spatial field. 

A 2D metal nano-disk structure MS-based plasmonic perfect absorber (PPA) with an absorbance of over 99% at 1050 nm and sensitivity of roughly 560 nm/R.I.U is presented in [118]. All-dielectric or all-metal MSs are preferable for PPAs owing to the easy model in arrangement and simple handling in manufacture, in contrast to traditional MMs whose capabilities depend on complex forms. The currently suggested MS-based PPAs for RI sensing, however, typically have a single band and a low sensitivity. Therefore, creating a brand-new multi-spectrum PPA for RI sensing applications is always highly desirable. It has been suggested and statistically analyzed to use an all-metal nanostructure MS-based quad-band PPA for RI sensing [119]. The suggested PPA has an assembly array of vertical split rings and many cylinders composed of an all-metal nanostructure. SPRs and guided modes combine to form the hybrid modes that primarily cause the quad-band perfect absorptions. It is thought that the suggested PPA could be used as a RI sensor because of its greater Q-factor. One example of this is the high sensitivity of a developed PPA-based sensor, which is about 4367 nm/R.I.U, 2162 nm/R.I.U, 1059 nm/R.I.U, and 908 nm/R.I.U, respectively. Due to its outstanding performance, the suggested quad-band PPA may find use in infrared spectroscopy, sensing, and detection [119].

**Figure 5:**
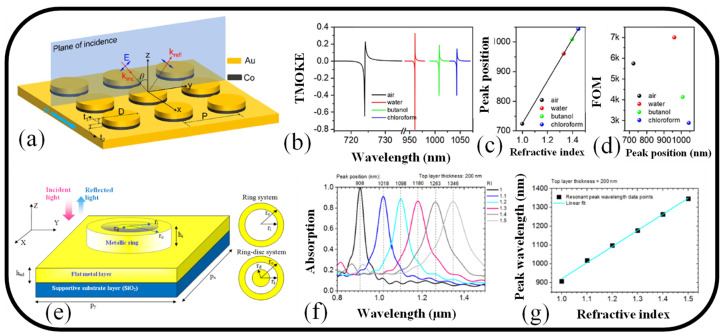
(**a**) Graphical interpretation of a magneto-plasmonic sensor established on a nanodisk array [82], (**b**) TMOKE spectrum for various ambient refractive indices [82], (**c**) Peak position of TMOKE curves as a function of RI [82], and (**d**) FOM as a function of wavelength [82]. (**e**) Graphical illustration of a plasmonic MS in 3D view. Reprinted with permission from Ref. [120], (**f**) Absorption spectrum. Reprinted with permission from Ref. [120], and (**g**) Plot of wavelength shift against ambient RI. Reprinted with permission from Ref. [120].

The fabrication of a periodic square array of Au/Co bilayer nano-disks on an optically thick metallic substrate results in the demonstration of a high-performance magnetoplasmonic sensor [82]. This results in the formation of an ultranarrow SPR mode with a full width at half maximum of 7 nm and RI sensitivity as high as 717 nm/R.I.U, whose high-quality resonance characteristic is like the surface lattice resonance linked to the far-field coupling between nano-objects. The schematic representation of the all-metallic MS sensor is illustrated in Figure 5a. Figure 5b illustrates how the TMOKE spectrum changes when a nanosensor is placed in various mediums. It can be observed that the system’s great sensitivity causes the MO signal to redshift dramatically as RI rises. Figure 5c illustrates a linear relationship between the resonance position and the RI by plotting the peak positions for each TMOKE spectrum as functions of RI [82]. The FOM of the magneto-plasmonic sensor in various mediums is illustrated in Figure 5d. In a water environment, the nanosensor impressively achieves a sky-high FOM close to 7000, which is about two orders of magnitude greater than that of gold- or silver-based nanosensors. It is important to note that a unique combination of localized and propagating SPRs on diffractive orders in periodic arrays constitutes the physical mechanism of the ultranarrow resonance in all-metallic nanostructures. The proposed nanosystem gains a massive transverse magneto-optical Kerr effect (TMOKE) value up to 0.65 generated by the hybrid plasmon mode in a dielectric medium with a refractive index of n = 1 because of the excellent SPR effect. It is theoretically demonstrated that the novel magneto-optical (MO)-SPR sensor proposed exhibits ultrahigh bulk RI sensitivities and incomparable sensing figures of merits (FOM) in a wide range of refractive indices thanks to the large EM field augmentation in the all-metallic nanostructure and the ultranarrow linewidth of the TMOKE spectrum [82].

It is suggested to use subwavelength rings and ring-discs on a flat metal substrate to create all-metal NIR plasmonic ideal absorbers as illustrated in Figure 5e [120]. Multi-resonant absorption is accomplished using a straightforward model principle. The single absorption mode is changed to dual absorption mode by passively adjusting the disc size. Furthermore, by enclosing the assemblies in a dielectric medium with a high dielectric constant, numerous resonant absorptions at tunable NIR wavelengths are accomplished. The absorption spectrum versus wavelength is plotted in Figure 5f. The wavelength shifts vs RI are displayed in Figure 5g to confirm the linearity. The data is linearly fit, and the best fit line was determined using the sensitivity parameter S = 857 nm/R.I.U. The proposed structure’s robust architecture makes it appropriate for optoelectronic components [120].

### 5.2. All-Dielectric MS-Based Sensors

All-dielectric MSs currently represent a sizable subgroup of MSs [121,122]. One of their characteristics is a sequence of Mie resonances created in nanostructures with high refractive indices [123,124]. As a result, these MSs were discovered to be physically rich. To enable E- and H-dipole responses established on Mie resonances, the unit structure for dielectric MSs is built with high RI dielectric resonators, such as silicon, germanium, or tellurium [125]. Both the H-dipole (first Mie resonance) and E-dipole resonances are activated when a dielectric particle is irradiated by a light wave whose frequency is below or close to the bandgap frequency of the material of the particle. The H and E Mie resonances can thus, at optical frequencies, boost the magnetic and electric fields at the particle’s core, and this amplification is connected to the fundamental characteristics of dielectric particles. As a result, the reactions of plasmonic and dielectric MSs are all pertinent to the properties of the unit structure, including its size and composition. By carefully planning the dimension parameters of each unit in the MSs, it is possible to control the electromagnetic field of the light wave that is dispersed by them.

Based on the dielectric MSs, more diversified optical functions, including phase-and-polarization control [126], flat lenses [127,128], and holograms [129], have been realized. The Ohmic loss that arises for conventional metallic materials makes it difficult to increase the performance and sensitivity of metallic sensors [130]. It is suggested to use graphene with an ordered arrangement of asymmetric Si nanorod units to create an RI sensor. The dielectric resonant nanostructures’ gaps and an asymmetric pair of nanorods are etched to create the sensor. As a result, a large portion of the EM waves was concentrated into hot spots at the nanoscale inside the gaps. The sensor’s sensitivity grew from 430 nm/R.I.U to 595 nm/R.I.U, and its FOM went from 956 R.I.U^−1^ to 4577 R.I.U^−1^, nearly a fivefold increase [130].

All-dielectric MSs made of high RI dielectric materials have just begun to gain traction as a new hotspot in micro/nanophotonics [81,104,131]. Dielectric nanophotonic structures localize light in the 10 nm range, which creates a zone of potent EM fields, in contrast to plasmonic MSs [132]. This event commonly referred to as a "hot spot," causes the local field to become 3–4 orders of magnitude more intense, which is favorable for increasing the sensitivity of analyte molecule detection. It is important to note that dielectric nanostructures have several advantages over metal (plasmonic) nanoparticle systems, the key one being compatibility with CMOS technology. Such dielectric nano-resonator-based sensors offer a different and potentially effective platform for biosensing and surface-enhanced spectroscopy [133]. A growing number of nano-dielectric materials have been developed and employed in recent years due to the continual advancement of nanoparticle production technology. In particular, titanium dioxide is a good substitute for metallic materials because it almost eliminates absorption loss in the visual region and supports EM resonance modes.

Numerous all-dielectric MS sensors have been exhibited in recent years for label-free optical sensing. In general, the sensing techniques can be divided into chiral molecule sensing [134], surface-enhanced spectroscopy, and refractometric detection. The all-dielectric MS is covered with analytes for refractometric detection. The resonant frequency shift required for molecule identification is caused by the binding of the surrounding media to the MS’s resonators, which results in localized changes in the RI. While the other techniques for vibrational spectroscopy and biomolecule sensing, surface-enhanced Raman spectroscopy (SERS) [135,136] and surface-enhanced infrared absorption spectroscopy [137,138], not only enable the detection of surface-bound molecules but also provide chemically specific information about each analyte.

To generate exceptionally surface-sensitive and spectrally sharp resonances for nanophotonic biosensors, symmetry-protected quasi-bound states in the continuum (BIC) driven by MSs with broken in-plane equilibrium are commonly employed [139]. Through the creation of Si-based asymmetric nanobar sets, a quasi-BIC mode with a dominating toroidal dipole (TD) and electric quadrupole (EQ) resonant characteristic in the NIR is stimulated, and this mode demonstrates ultrahigh sensitivity in the refractometric tracking of the local changing environment. The TD-EQ quasi-BIC mode displays strong and rigorously limited optical fields at the surface of tilted nanobar sets, and its RI susceptibility can be greatly improved for bigger aspect ratio nanopillars. This is in contrast to the usual E and H Mie-type dielectric resonator resonances, which have the enhanced field mostly within the resonator volume. The experimentally determined (simulated) sensitivity and FOM for nanobar sets with a height of 450 nm are 608 nm/R.I.U and 46 (612 nm/R.I.U and 85), respectively [139]. The CMOS-compatible approach may be utilized to develop an ultrahigh-sensitive all-dielectric platform suitable for on-chip integration and sensing device reduction in a wide range of medical diagnostics.

A 2D-periodic TiO_2_ dielectric grating structure with a guided mode is proposed as a high-sensitivity RI sensing device [140]. The improved nanosensing device can excite guided-mode resonance with an ultra-narrow linewidth of 0.19 nm, according to the results of numerical simulations. The sensitivity, Q factor, and FOM values of the nanosensing device can approach 82.29 nm/R.I.U, 3207.9, and 433.1, respectively, at a biological layer thickness of 20 nm. Additionally, the device exhibits good tolerance for the angle of incident light and is insensitive to polarization. This shows that using low-loss all-dielectric MSs to produce ultra-sensitive biosensor detection is a good approach [140].

**Figure 6:**
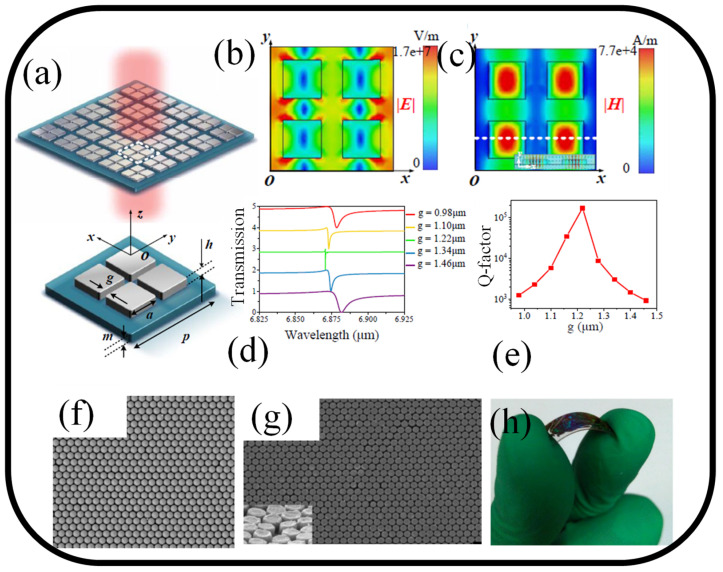
(**a**) Schematic representation of the symmetric MS [133], (**b**) E-field distribution [133], (**c**) Magnetic field distribution [133], (**d**) transmission spectrum of the MS [133], (**e**) Q-factor [133]. (**f**) SEM images of (**f**) PS spheres that self-assembled [141], (**g**) Si cylinders that were produced using RIEs. Both exhibit a regular hexagonal lattice. The tilted view (60°) of a specifically selected faulty area is represented in the inset in (**g**) to better illustrate the spatial morphology [141], and (**h**) the final MS sample shows its adaptability [141].

It is shown that all-dielectric MS-based high Q-factor sensing devices can detect small changes in the incidence angle and the media’s RI [133]. An all-dielectric MS established on a symmetric tetramer can function as an exceptional sensing platform for trace-amount molecules like protein A/G, 2, 4-DNT, and 2D material graphene with huge absorbance enhancement in the MIR broadband spectrum by using the light incident angular scanning method. The findings show that the envelope of absorbance amplitudes is in good accord with the vibrational mode of molecules and that absorbance enhancement factors in the MIR range between 5.75 and 6.80 μm can get as high as 10 dB. Figure 6a shows the suggested symmetric tetramer MS’s schematic diagram. In RI sensing, the suggested MS’s sharp Fano resonances are advantageous. The E- and H-field distributions of the unit cell at the resonance wavelength of 6.87 μm are calculated and are depicted in Figure 6b,c, respectively. This allows the resonance mechanism to be analyzed intuitively [133]. Figure 6d,e shows the transmission spectra and Q-factor for various gap sizes, respectively. The resonant wavelength is found to perform a blueshift when the gap (g) between the meta-atoms rises from 0.98 μm to 1.22 μm and performs a redshift when the gap (g) increases even higher from 1.22 μm to 1.46 μm [133].

The possibility for a flexible, all-dielectric MS created via nanosphere lithography (NSL) is presented, along with examples of how it might be used in sensing applications [141]. By monitoring transmission spectra, Si cylinders with a hexagonal lattice that are regularly spaced and made on a flexible polyethylene terephthalate (PET) substrate are used to detect applied strain and the surface dielectric environment. Additional computational simulations and experimental results agree. The coupled magnetic Mie resonance between closely packed Si cylinders is what causes the transmission peak. These very flexible Mie resonance-based sensing devices offer an alternative to conventional plasmon resonance-based sensing devices for sensing environmental fluctuations and give designers more options for creating photonic devices that operate in the optical regime. Figure 6f shows an SEM image of a self-assembled mask made of tightly packed monolayer polystyrene (PS) spheres. A single PS sphere has an estimated diameter of 350 nm on average. It is discovered that a brief period of isotropic O_2_ plasma etching, followed by another reactive ion etching (RIE) step, efficiently trims the margins of spheres, thoroughly etching the exposed Si layer. Figure 6f displays the SEM image of the Si cylinders’ planer hexagonal lattice. To better highlight the spatial morphology, a tiny defective area is chosen, as seen in the inset of Figure 6g, where the MS sample is tilted at around 60°. A single Si cylinder is thought to have an average diameter and height of 330 nm and 170 nm, respectively. The MS sample’s extraordinary flexibility is seen in Figure 6h. The PET substrate is around 180 μm thick. According to interference color, patterns are created in compact arrays, supporting SEM observations.

### 5.3. Hybrid MS-Based Sensors

At the nanoscale, light may be absorbed, scattered, and shaped in various ways by plasmonic MSs built from collections of dispersed metallic nanostructures. Combining plasmonic MSs with different materials to create hybrid versions creates new research opportunities and creative uses. The promise of both pure metallic and all-dielectric characteristics is concurrently exploited by efficient hybrid plasmonic-photonic MSs, which is highlighted as an emerging technology in flat optics [142,143]. Due to their ability to combine the strongest field enhancement of plasmonic metals with numerous low-loss radiation channels of dielectric resonators, hybrid metal-dielectric nanostructures have become increasingly popular [37,76,144,145,146,147,148]. 

**Figure 7:**
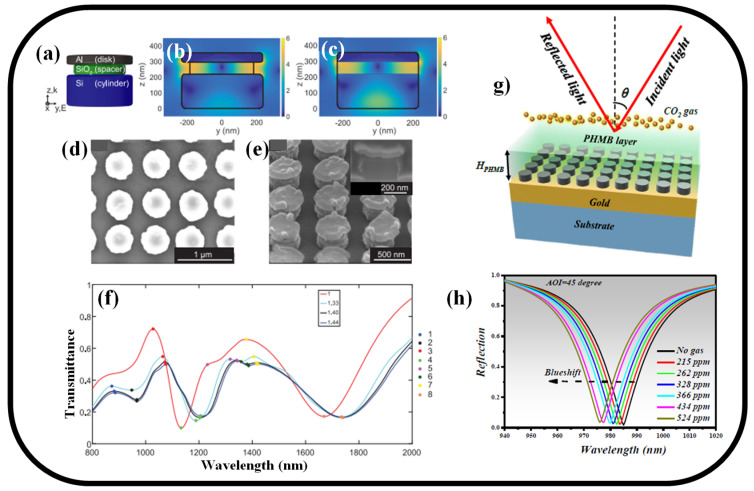
(**a**) The hybrid nanoantenna’s shape with its 50 nm SiO_2_ undercut. E-field amplitude enhancement surface plots (**b**) with and (**c**) without a 50 nm undercut in the SiO_2_. Reprinted with permission from Ref. [144] (**d**) A top view SEM photo of a hybrid nanoantenna array with a 670 nm period. Reprinted with permission from Ref. [144], (**e**) SEM picture of the hybrid nanoantenna array, tilted 52° to show the undercut in the SiO_2_ spacer. The cross-section of a single nanoantenna created via FIB is seen in the inset. Reprinted with permission from Ref. [144], (**f**) Experimental transmittance for four distinct background circumstances for bulk RI detection using a hybrid nanoantenna with a 50 nm undercut in the SiO_2_. Reprinted with permission from Ref. [144], (**g**) Schematic representation of hybrid MS covered with PHMB layer for gas sensing application [83], (**h**) Reflection spectrum versus CO_2_ gas concentration [83].

With a sensitivity of 208 nm/R.I.U, a successful array of these hybrid nanoantennas is created over a sizable area and used to detect bulk RIs [144]. Each nanoantenna consists of an Al disk and a Si cylinder joined together by a SiO_2_ spacer. The multipoles supported by these component parts and their mutual coupling are used to thoroughly investigate the nanoantenna’s optical response. An undercut in the spacer area is suggested to give the analyte access to the large EM field region as represented in Figure 7a. The E-field distribution in the hybrid nanoantenna unit with and without undercut in the SiO_2_ region is represented in Figure 7b,c, respectively. Figure 7d,e present the SEM images of the hybrid nanoantenna array with a period of 670 nm. Figure 7f shows the experimental transmittance for bulk RI sensing. To further improve the interaction of the E-field with the background medium and increase the nanoantenna’s sensitivity to 245 nm/R.I.U, an experimental undercut in the SiO_2_ region was added [144].

A hybrid MS-based perfect absorber that exhibits near-unity absorbance and can function as an RI sensing device is suggested [37]. Amorphous silicon (a-Si) nanodisk arrays are placed on top of a gold mirror, which is utilized to block transmission and activate the surface plasmon by scattering light across it at normal incidence. The suggested absorber has a maximum absorption of 99.8% at a 932 nm wavelength in the air medium and is polarization-independent. With a redshift in the resonant wavelength, the suggested absorber may sustain absorption at more than 99.7% when the environment’s RIs are changed from 1.33 to 1.41, considering real-world applications. The absorber exhibits near-unity absorbance over the RI range of 1.33 to 1.41 and a zero-reflectance property at a particular wavelength because of the electric and magnetic dipoles’ impedance matching. This characteristic might be used as a plasmonic sensing device to determine the RI of the immediate environment. Over the sensing range of 1.33 to 1.41, the suggested plasmonic sensing device exhibits an average sensitivity of 325 nm/R.I.U and a maximum sensitivity of 350 nm/R.I.U. The proposed MS has potential uses in organic and biochemical detection, solar photovoltaic and photodetectors, and related fields [37].

A hybrid MS perfect absorber (HMSPA) established on square meta-atoms (S-MAs) and hollow-square meta-atoms (HS-MAs) is described for its RI and temperature sensing capabilities [76]. Both models are also suitable for filtering applications since they can deliver >90% absorption in the narrowband area. Compared to S-MAs, the HMSPA with HS-MAs is far more sensitive to minute changes in the RI of the surrounding medium, making it the perfect choice for biosensing applications. The S-MA-based HMSPA’s sensitivity is around 135 nm/R.I.U and can be increased to 355 nm/R.I.U by employing HS-MAs. Additionally, using the suggested device for temperature sensing is made possible by placing a material that measures temperature on the surface of the MS. A temperature sensitivity of −0.18 nm/°C is achieved for the temperature range between 20 °C and 60 °C for the HS-MA-based HMSPA due to the remarkable thermo-optic coefficient of polydimethylsiloxane. The suggested HMSPA devices provide easy sensor fabrication and light coupling capabilities, making them potentially helpful in filtering, biosensing, and temperature-sensing applications [76].

By applying a thin layer of functional host material—a polyhexamethylene biguanide polymer to the MS, a CO_2_ gas sensing application is investigated [83]. The schematic of the MS-based gas sensing device is represented in Figure 7g. Due to the CO_2_ gas being absorbed, the host material’s RI decreases. The resonance wavelength of the ideal absorber consequently exhibits a remarkable blueshift as represented in Figure 7h. The suggested sensing device model, which is established on MS, was used to detect CO_2_ gas concentrations in the range of 0–524 ppm. A gas concentration of 434 ppm yielded a maximum sensitivity of 17.3 pm/ppm. 

## 6. Prospects and Challenges

Most of the current work has been focused on developing a single optical capability on a single layer of MSs. MSs, which are discrete optical elements stacked in arrays of nanostructures, present a rare chance to interleave many functionalities in a single layer, which is not feasible with traditional optics [149]. To make quantum circuits more useful, this technique might be used, for instance, to create spin-dependent MSs and to regulate the phase of a quantum emission. Here, new methods for designing MSs that consider symmetry, topology, and disorder are required. To increase the Q-factors of dielectric MS from their existing state, precise design techniques are also needed. Finally, the number of devices on a chip may be scaled up from tens to thousands by precisely aligning numerous MSs vertically. The MS array may also be enhanced with surface functionalization to produce chemically specific, ultra-sensitive biosensing methods without the need for the complicated apparatus now needed.

Metallic and dielectric materials are frequently seen in MSs and MMs. Dielectrics can have a positive dielectric constant (permittivity) in the 1–10 level in the optical range and be loss-free. Nevertheless, losses that are typically associated with metallic components might seriously impair the functionality of a metamaterial system. Noble metals like gold and silver are typically employed as metal materials in MM systems because of their relatively reduced losses. For effective optical-range applications, the losses are still too great, even in these metals. The fact that metals have too high and untunable negative permittivity magnitudes is another issue. The metal component’s permittivity should be low and of the same order as the dielectric component for the best MM designs. However, in the optical range, metals have permittivity values on the order of −100. As a result, many applications for MMs do not fit normal metals and require alternate plasmonic materials.

It is also necessary to create low-cost, large-area manufacturing methods to move away from the pricy electron beam lithography (EBL) that is now used. To create nanoscale structures, EBL employs a concentrated e-beam wavelength at high accelerated voltages. The EBL process involves exposing a high-energy e-beam to an e-beam resist, such as PMMA, much like conventional photolithographs do when ultraviolet (UV) light is applied to a photo-resistant substrate. As a result, the organic structure becomes divided, and the segment exposed to the e-beam completely dissolves in the developer solution. Following the deposition of metal or dielectric layers, the resist is removed to reveal the nanoscale patterns. This method uses the "lift-off" procedure, thus the reverse design specified by the EBL must be the necessary pattern. EBL is ideally suited and doesn’t need a physical mask for pattern transfers to produce nanoscale details below the diffraction barrier of conventional photolithographic methods. Subwavelength resonators are created using the EBL technology. The creation of high-performance large-scale MMs must overcome three fundamental limitations, namely, the lengthy writing durations since the technology is serial; the frequent stitch flaws; and the lack of stability and probable astigmatism of the e-beam. Additionally, serial patterning results in drifting beam instability, a large region of several steps resulting in low resolution and stitching flaws [150,151]. The capacity of the designs to be reproduced without a significant section of misalignment is also negatively impacted by stitching faults. Finally, the effectiveness of the approach is significantly influenced by the consistency, accuracy, and reputation of e-beam whitening. An outer voltage source called a blanket beam is utilized to turn on and off e-beams while shifting nanoscale functionality. Any change throughout the lengthy writing durations typically involved in this technique may result in incoherent exposure and cause geometric inaccuracies [152].

Nanoimprint lithography (NIL) technology is a way to achieve high throughput and resolution while also having the benefits of large-area patterning and low cost [153]. A patterned master mold, typically created via the EBL method, is needed for the first phase of NIL, which replicates substantially more quickly than EBL once a mold has been created. NIL is furthermore compatible with the existing panel industry production method. So, it substantially boosts the feasibility of commercial mass production of MS devices [154]. Table 1 summarizes the sensing capabilities of a few of the recently proposed all-dielectric, all-metallic, and hybrid MS structures.

## 7. Conclusions

MSs have widened the horizons of numerous research fields. Due to their high sensitivity and selective biomarker detection and measurement capabilities—which are primarily used for precise and early illness conditions diagnosis—sensors established on these artificially made materials have a distinct advantage in the sensing area. In this review, three vital formations of MS-based perfect absorbers are evaluated which can be employed for sensing applications. Plasmonic (all-metallic or hybrid) MSs are made of novel metals, and by adjusting their optical and geometric features, it will be possible to identify biomarkers and biomolecules very accurately. Such terahertz/gigahertz (THz/GHz) structures are well suited for label-free and contactless detection of various bacteria, viruses, and cancer biomarkers. Localized surface plasmon resonance (LSPR), which results from the collective oscillations of electrons in a metal, occurs in plasmonic MSs. Plasmonic MSs benefit from the capacity to detect analytes directly at the metal surface, where the field is tightly constrained. The spectral response is significantly changed by the enhanced light-matter interaction with the analyte caused by this extreme field confinement. Conversely, metals provide high Joule heating, which might change an analyte’s properties. Additionally, a resonator with significant dissipation may also have a low Q-factor. The energy stored in the resonator concerning the energy lost by radiation or Joule heating is measured by the Q-factor. The detection sensitivity is restricted by a low Q-factor. MSs are created utilizing dielectric nanoparticles that support both electric and magnetic modes established on the Mie theory to address the loss problem. Due to the lack of Joule heating, dielectric MSs have a higher Q-factor than plasmonic MSs. Nevertheless, the modes supported by dielectric meta-atoms have a larger mode volume and are less localized. If a high analyte volume is being employed, all-dielectric MSs may be helpful for sensing applications.

## Figures and Tables

**Table 1:** Performance comparison of all-dielectric, all-metallic, and hybrid-MS-based sensors.

Type of MS	Application	Materials	Sensitivity	FOM	Reference
Hybrid	Biosensing	Si, SiO_2_, Au	460–492 nm/R.I.U	76.7–82 R.I.U^−1^	[72]
All-dielectric	Biosensing	Si, SiO_2_	1.43 μm/R.I.U	-	[133]
Hybrid	Gas sensing	Si, Au, PHMB	17.3 pm/ppm	-	[83]
All-dielectric	Biosensing	Si, SiO_2_	155 nm/R.I.U	387,000 R.I.U^−1^	[103]
All-metallic	Biosensing	Au	>850 nm/R.I.U	-	[120]
Hybrid	Temperature and Biosensing	Si, Au, PDMS	−0.18 nm/°C and 355 nm/R.I.U	-	[76]
All-metallic	Biosensing	Au, Co	717 nm/R.I.U	7000 R.I.U^−1^	[82]
All-metallic	Biosensing	-	700 GHz/R.I.U	0.6 R.I.U^−1^	[155]
Hybrid	Biosensing	Si, SiO_2_, Au	350 nm/R.I.U	-	[37]
All-dielectric	Biosensing	PDMS, TiO_2_	-	-	[156]
Hybrid	Biosensing	Si, Au	25.3, 41.3 and 31.9 THz/R.I.U	-	[157]
All-dielectric	Temperature and Biosensing	InSb	5.9 GHz/K and 6.4 GHz/K, 1.3 THz/R.I.U and 1.0 THz/R.I.U	-	[158]
All-dielectric	Biosensing	Si, SiO_2_	500.75 nm/R.I.U	3013.33 R.I.U^−1^	[159]
All-dielectric	Biosensing	TiO_2_, SiO_2_	82.29 nm/R.I.U	433.1 R.I.U^−1^	[140]
Hybrid	Biosensing	Graphene, dielectric, gold	9.8°/R.I.U	-	[160]
All-metallic	Biosensing	Gold	4367 nm/R.I.U, 2162 nm/R.I.U, 1059 nm/R.I.U, 908 nm/R.I.U	-	[119]
All-dielectric	Biosensing	Silicon, Quart	70 nm/R.I.U	2970 R.I.U^−1^	[122]
All-dielectric	Biosensing	Silicon, Graphene	430 nm/R.I.U to 595 nm/R.I.U	956 R.I.U^−1^ to 4577 R.I.U^−1^	[130]
Hybrid	Biosensing	Silicon, Gold, SiO_2_	68.6 nm/R.I.U	3.5 R.I.U^−1^	[161]
Hybrid	Biosensing	Aluminium, CaF_2_, silicon	1800 nm/R.I.U	62 R.I.U^−1^	[70]
Hybrid	Biosensing	Silver, hBN, graphene	1.5458 × 10^9^ μm/R.I.U	-	[162]
Hybrid	Biosensing, temperature	LiNbO_3_, graphene, gold, quartz	981 nm/R.I.U, −0.23 nm/°C	61.31 R.I.U^−1^	[105]

## Data Availability

Not applicable.

## Abbreviations

Metamaterial = MM; Metasurface = MS; Electromagnetically induced transparency = EIT; Refractive index = RI; Quality factor = Q-factor; Figure of merit = FOM; Sensitivity = S; Surface plasmon polariton = SPR; Localized surface plasmon resonance = LSPR; Electron beam lithography = EBL; Nanoimprint lithography = NIL; Scanning electron microscopy = SEM; Mid-infrared = MIR; Focused ion beam = FIB; Refractive index unit = RIU; Electromagnetic = EM.
